# MLKL promotes cellular differentiation in myeloid leukemia by facilitating the release of G-CSF

**DOI:** 10.1038/s41418-021-00811-1

**Published:** 2021-06-02

**Authors:** Xin Wang, Uris Ros, Deepti Agrawal, Eva C. Keller, Julia Slotta-Huspenina, Veronika Dill, Bo Shen, Run Shi, Tobias Herold, Claus Belka, Ritu Mishra, Florian Bassermann, Ana J. Garcia-Saez, Philipp J. Jost

**Affiliations:** 1 Medical Department III for Hematology and Oncology, School of Medicine, Klinikum rechts der Isar, Technical University of Munich, Munich, Germany; 2 Department of Internal Oncology, Jiangsu Cancer Hospital (Nanjing Medical University Affiliated Cancer Hospital) and Jiangsu Institute of Cancer Research, Nanjing, China; 3 Institute for Genetics, Faculty of Mathematicmas and Natural Sciences, Universität zu Köln, Cologne, Germany; 4 Institute of Pathology, School of Medicine, Klinikum rechts der Isar, Technical University of Munich, Munich, Germany; 5 Department of Radiation Oncology, University Hospital, Ludwig-Maximilians University, Munich, Germany; 6 Laboratory for Leukemia Diagnostics, Department of Medicine III, University Hospital, LMU Munich, Munich, Germany; 7 Research Unit Apoptosis in Hematopoietic Stem Cells, Helmholtz Zentrum München, German Research Center for Environmental Health (HMGU), Munich, Germany; 8 German Consortium for Translational Cancer Research (DKTK) partner site TUM, German Cancer Research Center Heidelberg (DKFZ), Heidelberg, Germany; 9 Center for Translational Cancer Research, Translatum, Technical University of Munich, Munich, Germany; 10 Division of Clinical Oncology, Department of Medicine, Medical University of Graz, Graz, Austria

**Keywords:** Cancer, Cancer

## Abstract

The blockade of cellular differentiation represents a hallmark of acute myeloid leukemia (AML), which is largely attributed to the dysfunction of lineage-specific transcription factors controlling cellular differentiation. However, alternative mechanisms of cellular differentiation programs in AML remain largely unexplored. Here we report that mixed lineage kinase domain-like protein (MLKL) contributes to the cellular differentiation of transformed hematopoietic progenitor cells in AML. Using gene-targeted mice, we show that MLKL facilitates the release of granulocyte colony-stimulating factor (G-CSF) by controlling membrane permeabilization in leukemic cells. *Mlkl*^*−/−*^ hematopoietic stem and progenitor cells released reduced amounts of G-CSF while retaining their capacity for *CSF3* (G-CSF) mRNA expression, G-CSF protein translation, and G-CSF receptor signaling. MLKL associates with early endosomes and controls G-CSF release from intracellular storage by plasma membrane pore formation, whereas cell death remained unaffected by loss of MLKL. Of note, *MLKL* expression was significantly reduced in AML patients, specifically in those with a poor-risk AML subtype. Our data provide evidence that MLKL controls myeloid differentiation in AML by controlling the release of G-CSF from leukemic progenitor cells.

## Introduction

Acute myeloid leukemia (AML) is characterized by the accumulation of genetic aberrations in hematopoietic stem/progenitor cells [1, 2]. Genetic aberrations often result in the repression of myeloid differentiation and cell death as two critical hallmarks of AML [3, 4]. The differentiation blockade in AML is largely attributed to the loss of lineage-specific transcription factor function relevant for the progression of myeloid differentiation along specific lineages in hematopoiesis [1]. Disruption of transcription factor function is mostly mediated by chromosomal translocations, mutations, or transcriptional repression such as seen in the case of the leucine zipper CCAAT-enhancer-binding protein α (C/EBPα) [5].

Programmed cell death represses leukemogenesis by killing transformed cells and, in the case of inflammatory forms of cell death, also by eliciting an inflammatory response [4]. The inflammation results in the propagation of a myeloid differentiation program based, at least in part, on the release of interleukin-1ß (IL-1ß) from AML cells in response to oncogenic signaling effectively repressing leukemogenesis [6]. Cell death mediated by receptor interacting protein kinase 3 (RIPK3) has a critical tumor-suppressive function during leukemogenesis [6] mediated by the induction of cell death and the inflammation-driven myeloid differentiation of the AML stem/progenitors [4, 6].

Necroptotic cell death provides a substantial inflammatory stimulus, which is largely mediated by damage-associated molecular patterns (DAMPs) released from dying cells [7, 8] eventually resulting in the activation of an immune response [9]. Necroptosis has evolved as an innate immunity mechanism against viral infection [10–12]. Central mediators of necroptotic cell death are RIPK3 and its downstream effector mixed lineage kinase domain-like protein (MLKL). Upon upstream activation, RIPK3 phosphorylates MLKL within the pseudo-kinase domain resulting in the unleashing of the N-terminal four-helix bundle domain (4HB) of MLKL [13]. Alternatively, kinases from the TAM family (TYRO3, AXL, and MER) have been reported to also phosphorylate MLKL to promote necroptosis [14]. This activated form of MLKL then translocates to the membrane, oligomerizes, and forms pores thereby mediating the release of cytoplasmic content into the extracellular space [13, 15–23].

MLKL activation and translocation to the plasma membrane represents the final stage of the necroptotic pathway [24]. Yet, MLKL has also been linked to different intracellular processes including regulation of inflammatory cytokines and endosomal trafficking of membrane-associated proteins. Yoon et al. reported that MLKL contributed to the endosomal trafficking of membrane-associated proteins such as the epithelial growth factor receptor (EGFR) in a necroptosis-independent function [25].

Here, we report that MLKL mediates the release of granulocyte colony-stimulating factor (G-CSF) via controlling plasma membrane permeabilization, thereby effectively promoting myeloid progenitor differentiation during conditions of inflammation as well as leukemogenesis.

## Methods

### Mouse lines, cells, plasmids

The *MLKL*^−*/−*^ mice have been described previously [13] and were used at 8–9 weeks of age for bone marrow (BM) cell collection from at least three mice of the same age for each experiment [6]. Animals were maintained under specific pathogen-free conditions, and all animal experiments were approved by the District Government of Upper Bavaria. The primary BM cells were harvested from mice of indicated genotypes 4 days after injection of 150 mg/kg 5-fluorouracil (5-FU; Sigma) intraperitoneally and cultured in RPMI medium containing 20% FCS (Fetal Calf Serum) supplemented with growth factors (10 ng/ml IL-3, 10 ng/ml IL-6; R&D Systems, 100 ng/ml SCF; eBioscience). Briefly, for producing BM-derived macrophages (BMDMs), the BM cells were treated with 40 ng/ml M-CSF (Biolegend^®^ 576408) for 7 days; after that cells were detached with 0.5 mM EDTA/PBS for about 2 min on ice for reseeding. To block membrane pore formation, polyethyleneglycols (PEGs, Sigma) with different molecular weights (PEG2000, PEG3000, PEG4000, PEG5000, PEG6000, PEG8000) were diluted in the medium with a final concentration at 100 mM. One day after treatment the cells and supernatant were harvested for further analysis.

### Colony formation assay

For colony formation assays, all duplicate cultures were performed in 35 mm Petri dishes with MethoCult^TM^ GF M3434 medium (STEMCELL Technologies Inc.). GFP^+^/Lin^−^ (green fluorescent protein positive/lineage negative) BM cells were sorted and seeded at a density of 2500 cells/plate and after 10–14 days the colonies were counted by light microscopy. The FMS-like tyrosine kinase 3-internal tandem duplication (FLT3-ITD), mixed lineage leukemia-eleven nineteen leukemia translocation (MLL-ENL), and AML-ETO (RUNX1/RUNX1T1 translocation) retroviral plasmids have been previously described [6]. Retroviral supernatants were produced in the packaging cell line Phoenix by using Metafectene^TM^ Pro transfection, and the pMIG empty plasmid served as a control [6].

### Flow cytometry (FACS)

Standard FACS staining protocol was followed as previously described [6]. In short, cells were washed and resuspended at a concentration of 1 × 10^6^ cells/ml. Then cells were pre-incubated with Fc-block and subsequently stained with fluorescently labeled antibodies as listed in the antibody list (Table S4). Propidium iodide (PI; Invitrogen) staining was used for viability gating. Flow analysis was performed on a BD FACS Canto II (BD Biosciences) and for GFP sorting, a BD FACSAria™ III cell sorter was used; data were analyzed using FlowJo^TM^. For intracellular staining, the cells were fixed with 4% paraformaldehyde (pH = 7) in PBS (room temperature, 10 min), then permeabilized with 0.1% Triton-X100 for 5 min in room temperature and blocked with 3% FBS. Cells were incubated 1 h with the indicated primary and secondary antibodies. The supernatant cytokine measurement cytometric bead array (CBA; BD Biosciences) was used according to the manufacturer’s instructions. Each assay was performed in triplicate.

### Fluorescence confocal microscopy

The WT or *MLKL*^−*/−*^ BM cells were seeded in the chamber slide in the density of 1 × 10^6^/ml for 2 days (with cytokine cocktail, 10 ng/ml IL-3, 10 ng/ml IL-6; R&D Systems, 100 ng/ml SCF; eBioscience), for immunofluorescence staining, the cells were fixed with 4% paraformaldehyde (pH = 7) in PBS in room temperature for 10 min, then permeabilized with 0.1% Triton-X100 for 5 min in room temperature and blocked with 3% FBS. After that cells were incubated for 1 h with the indicated antibodies and then with secondary antibodies (Table S4). Images of the immune-stained cells were captured with a white light laser confocal microscope (TCS SP8 X, Leica Microsystems). The co-localization between EEA1 and G-CSF was calculated with Leica Application Suite X software (Ver.3.4.2, Leica).

### RNA isolation and real-time PCR

RNA isolation was performed by Nucleospin^®^RNA (MACHEREY-NAGEL), following the manufacturer’s instructions. RNA concentration and purity were determined using the NanoDrop spectrophotometer (NanoDrop Technologies). One µg total RNA was reverse transcribed to 20 µl cDNA by SuperScript II Reverse Transcriptase (Life Technologies), The qPCR was performed using LightCycler^®^ 480 (Roche) Real-Time PCR System, the reaction protocol was 95 °C for 10 min, followed by 40 cycles of 92 °C for 15 s and 62.5 °C for 1 min. Every sample was in triplicate and normalized to the reference gene expression using the 2^−ΔΔCt^ method, all the primers in Table S3.

### Immunohistochemistry (IHC)

Primary AML samples were obtained from patients treated at the III. Medical Department at the Technical University of Munich after approval of the local ethics committee (approval no. 62/16S from February 10, 2016 to 2790/10 from April 30, 2010). Informed consent was obtained from patients at study entry. For IHC eligible patients had received a diagnosis of de novo AML, which had been confirmed by means of a cytologic examination of blood and BM. Cases were re-evaluated using hematoxylin and eosin (H&E), Giemsa, AS-D chloroacetate esterase and standard diagnostic IHC (CD34, CD117, MPO). The infiltration rate of AML blasts was >50% in most cases. Biopsies were fixed in 4% formaldehyde, EDTA decalcified, and paraffin-embedded. IHC was performed using an automated immunostainer with the VIEW DAB detection kit (Ventana Medical System, Roche) according to the company’s protocols for open procedures. Biopsies were stained with anti-MLKL (EPR17514, Abcam, 1:500) according to the manufacturer’s instructions and counterstained with hematoxylin. Appropriate positive controls were used to confirm the adequacy of the staining. Specific MLKL staining was localized to the cytoplasm. Human BM samples were collected according to the institutional guidelines and written informed consent was obtained from all patients in accordance with the Declaration of Helsinki. All use of patient material was approved by the Local Ethics Committees.

### Statistical analysis and data source

Data are presented as the mean ± SD or SEM and analyzed using a Student’s *t*-test (two-tailed) or one-way ANOVA with posthoc (Dunnett’s multiple comparisons test) analysis. And *P < 0.05, **P < 0.005, ***P < 0.0005 were calculated by SPSS Statistics (version 20.0, IBM Corp.) or GraphPad Prism (version 7.05, GraphPad Software, Inc.). The level 3 Cancer Genome Atlas (TCGA) acute myeloid leukemia (LAML) datasets containing 173 patient samples were downloaded for analysis, where “count” refers to RNA-Seq by expectation-maximization (RSME)-normalized transcript read count [26]. The unit for gene expression is “log2(count + 1)” [27]. The GSE13204, GSE15061, and GSE37642 were downloaded from gene expression omnibus (GEO) [28–32]. For gene set enrichment analysis (GSEA) and the heatmap, the latest official tool was downloaded from http://software.broadinstitute.org/gsea (Version 3.0) [33].

## Results

### AML patients show significantly reduced MLKL expression

**Fig. 1:**
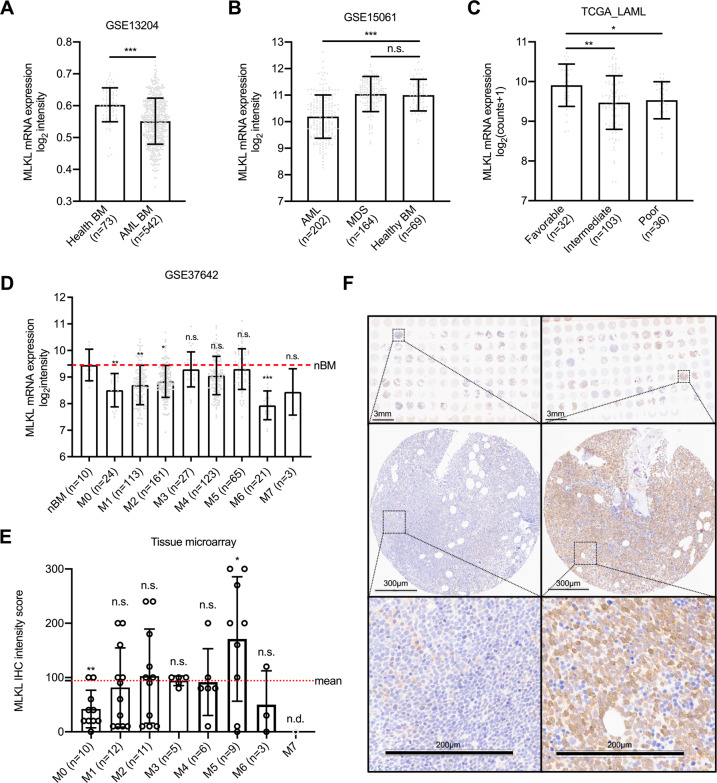
Significantly reduced *MLKL* expression in AML patients. **A** Data from GSE13204 (GPL570 platform) indicate *MLKL* expression in AML samples (all genetic or cytogenetic subtypes) compared to healthy controls (*t*-test, mean with SD). **B** Data from GSE15061 indicate *MLKL* expression in MDS and healthy controls compared to AML patients (one-way ANOVA *P* < 0.0005 with Dunnett’s multiple comparisons test, mean with SD). **C** Data from the TCGA AML cohort (TCGA_LAML) categorized by clinical risk category (one-way ANOVA *P* < 0.005 with Dunnett’s multiple comparisons test, mean with SD). **D** Data from GSE37642 display the *MLKL* expression in different AML subtypes based on FAB system (one-way ANOVA *P* < 0.0005 with Dunnett’s multiple comparisons test (all the other groups compared to normal BM/nBM), mean with SD). **E** AML bone marrow samples from tissue microarray (TMA) were enrolled for MLKL immunohistochemistry (IHC) staining, shown are the MLKL IHC intensity data from TMA in different AML subtypes based on FAB system, and M7 group not done (n.d.) (*t*-test, all the other groups compared to the whole cohort mean values). **F** Representative images of immunohistochemistry of bone marrow biopsies from AML patients stained for MLKL (brown) and counterstained with hematoxylin (blue). MLKL-specific staining was localized to the cytoplasm. Scale bar as indicated in the pictures. *P < 0.05, **P < 0.005, ***P < 0.0005.

Based on the critical tumor-suppressive function of RIPK3 during leukemogenesis [6], we determined the role of its downstream effector MLKL in AML. We first characterized gene expression levels of *MLKL* in human AML samples in 542 human AML BM samples and 73 healthy bulk BM controls (GSE13204). We found that *MLKL* expression was significantly reduced in AML BM (across all genetic or cytogenetic aberrations) (Fig. 1A), which was in line with the MLKL expression in WHO-categorized AML as previously reported [6]. Moreover, we utilized an independent dataset (GSE15061) to corroborate these data and compared 202 AML patients to 164 samples from patients diagnosed with myelodysplastic syndromes (MDS) to 69 healthy control BM samples. Whereas *MLKL* expression remained unchanged between MDS (various clinical risk categories) and healthy controls, its expression was significantly repressed in AML patients (Fig. 1B).

We next explored the *MLKL* expression based on the clinical AML risk category. Subdividing patients from the TCGA AML dataset (TCGA-LAML) according to their clinical risk classification, we found that patients with a favorable risk (*n* = 32) had significantly higher *MLKL* expression compared to patients in the intermediate (*n* = 103) or poor (*n* = 36) risk (Fig. 1C). Of note, when patients were grouped according to the outdated French–American–British (FAB) classification system, *MLKL* expression was significantly lower in the most immature samples (FAB M0–M2) compared to more differentiated AML subgroups (FAB M3–M5) or BM samples from healthy individuals (Fig. 1D). This was confirmed in primary human AML BM specimens from our in-house AML cohort, which showed that the MLKL protein expression was reduced in the most immature samples (M0 by FAB) (Fig. 1E, F).

These data suggested that MLKL might serve as a tumor-suppressor in AML. Yet, screening of the TCGA AML dataset (*n* = 197) failed to show any coding mutations in the *MLKL* gene, only one mutation was found in the TCGA dataset for AML in the PanCancer cohort and no mutational hotspot was identified across other cancer types (Fig. S1A, B) [34, 35].

### MLKL promotes differentiation of myeloid leukemic stem and progenitor cells

Based on these findings, we postulated that loss of MLKL might benefit leukemia development or progression. To investigate this, we expressed several clinically relevant common AML driver oncogenes in primary murine hematopoietic stem and progenitor cells (HSPC) ex vivo. Upon retroviral transduction, we generated *Mlkl*^*−/−*^ or WT primary leukemic cells using AML-ETO, FLT3-ITD, or MLL-ENL as driving oncogenes. After 5 days of culture, we characterized the number of primitive myeloid cells defined as either lineage-negative (Lin^−^) myeloid cells or myeloid progenitor cells (Lin^−^ Sca1^−^ c-Kit^+^) (containing common myeloid progenitors [CMP]; granulocyte–macrophage progenitors [GMP]; and megakaryocyte–erythroid progenitors [MEP]) and observed no relevant difference in the viability of *Mlkl*^*−/−*^ or WT cells (Fig. S2A–C).

**Fig. 2:**
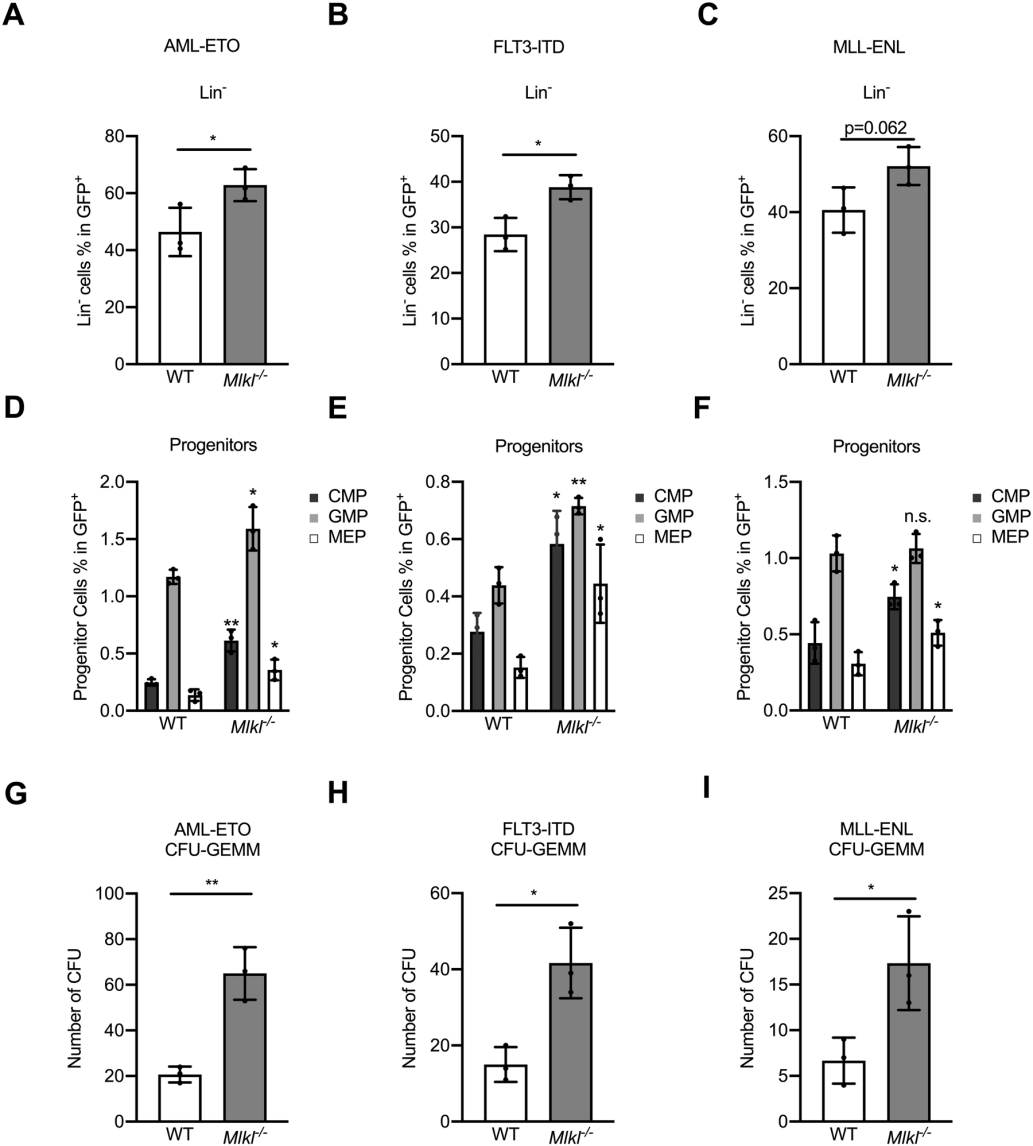
MLKL deletion restricts malignant myeloid differentiation. Five days after oncogene transduction the bone marrow cells were collected for the following measurement or colony formation assay. **A**–**C** Shown is the percentage of lineage negative cells (Lin^−^) in GFP^+^ cells (GFP^+^) of BM cells (WT and *Mlkl*^*−/−*^) transduced with AML-ETO, FLT3-ITD, or MLL-ENL (*t*-test, mean with SD compared to WT). Detailed FACS plots shown in Fig. S2D. **D**–**F** Shown is the percentage of progenitor cells in GFP^**+**^ cells (*t*-test, mean with SD compared to WT) detailed FACS plots in Fig. S2D). **G**–**I** Shown is the colony count of the myeloid progenitor population CFU-GEMM flow-sorted from *Mlkl*^*−/*−^ and WT GFP^+^/Lin^−^ BM cells after 10 days of culture in methocult media (*t*-test, mean with SD compared to WT). *P < 0.05, **P < 0.005, ***P < 0.0005.

Our data revealed that loss of MLKL resulted in an expansion of progenitor populations in primary murine BM (Figs. 2A–C, S2D), which was mainly driven by increased numbers of CMP implying that MLKL contributed to the myeloid differentiation of progenitor cells in AML (Figs. 2D–F, S2D).

To explore the possibility that the increased number of progenitors in *Mlkl*^*−/−*^ BM might be explained by the inability of *Mlkl*^*−/−*^ progenitors to differentiate into a specific myeloid lineage, we tested their colony-forming capacity. In line with the data obtained by FACS, *Mlkl*^*−/−*^ colonies showed an accumulation of the myeloid progenitor population as measured by an increase in the CFU-GEMM colonies (granulocyte, erythrocyte, monocyte, megakaryocyte progenitors) compared to WT colonies (Fig. 2G–I).

In summary, the loss of MLKL resulted in a marked expansion of primitive myeloid progenitor cells and primitive hematopoietic colonies, which suggested a contribution of MLKL to myeloid progenitor differentiation in leukemia.

### *MLKL* expression correlates with an inflammatory response signature in human AML

**Fig. 3:**
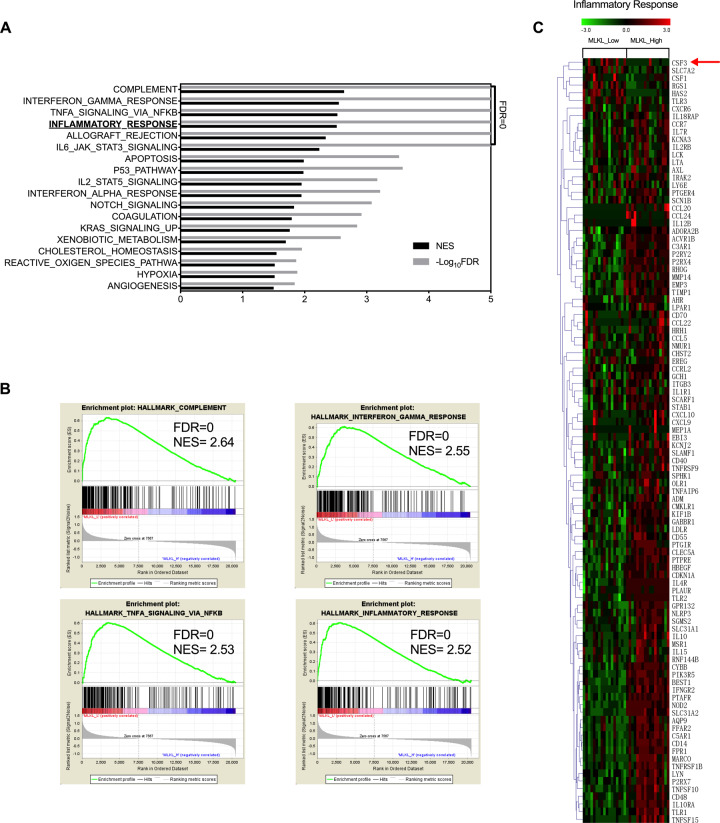
*MLKL* expression correlates with an inflammatory response signature in human AML. Shown are the top 10% *MLKL* high expression samples (*n* = 17) vs. the bottom 10% *MLKL*^low^ expression samples (*n* = 17) from 173 AML patients’ samples in TCGA_LAML dataset. These transcriptome data were submitted for GSEA (gene set enrichment analysis). **A** NES (normalized enrichment scores) with −log_10_FDR in significant pathways from GSEA analysis (the FDR of top 6 pathways are zero, thus their −log_10_FDR = ∞), the detailed data in Table 1. **B** Top 4 significant pathways from gene set enrichment analysis (GSEAs), including complement, interferon-γ, TNF, and inflammatory response (additional plots in Fig. S3A). **C** Heatmap of inflammatory response pathway comparing *MLKL*^high^ and *MLKL*^low^ samples.

We next explored the mechanism by which MLKL acts on cellular differentiation in the hematopoietic system. Using the TCGA AML dataset (TCGA_LAML) including 173 AML patient samples, we selected the samples with the top 10% highest and the top 10% lowest *MLKL* mRNA expression (17 samples in each group) [36]. We then submitted the transcriptome data of these samples for GSEA to distinguish differentially expressed gene signatures. The GSEA results showed a significant correlation between *MLKL* expression and several inflammatory response pathways (Fig. 3A). Specifically, we identified a significant correlation for TNF signaling, complement signaling, interferon-γ signaling, and the inflammatory response signature (Figs. 3B, S3A, Table 1).

**Table 1:** GSEA result.

Pathway name	ES	NES	FDR *q*-val	FWER *p*-val
COMPLEMENT	0.6324	2.6355	0	0
INTERFERON_GAMMA_RESPONSE	0.6137	2.5515	0	0
TNFA_SIGNALING_VIA_NFKB	0.6069	2.5288	0	0
INFLAMMATORY_RESPONSE	0.6091	2.5175	0	0
ALLOGRAFT_REJECTION	0.5611	2.3393	0	0
IL6_JAK_STAT3_SIGNALING	0.6032	2.2378	0	0
APOPTOSIS	0.4932	1.9857	0.0003	0.001
P53_PATHWAY	0.4766	1.9803	0.0003	0.001
IL2_STAT5_SIGNALING	0.4711	1.951	0.0007	0.003
INTERFERON_ALPHA_RESPONSE	0.5165	1.9497	0.0006	0.003
NOTCH_SIGNALING	0.5965	1.8301	0.0008	0.005
COAGULATION	0.4517	1.793	0.0012	0.008
KRAS_SIGNALING_UP	0.4225	1.7606	0.0014	0.01
XENOBIOTIC_METABOLISM	0.41	1.6977	0.0026	0.019
CHOLESTEROL_HOMEOSTASIS	0.4326	1.5466	0.0111	0.084
REACTIVE_OXIGEN_SPECIES_PATHWA	0.4574	1.519	0.0137	0.108

*ES* enrichment score, *NES* normalized enrichment score, *FDR* *q*-val false discovery rate *q*-value (adjusted *p*-value), FWER *p*-*val* family wise error rate *p*-value.

Moreover, we utilized the human gene expression data to identify genes most differentially expressed between AML patients with the highest *MLKL* expression (AML *MLKL*^high^) compared to AML patients with the lowest *MLKL* expression (AML *MLKL*^low^) (Table S1, Fig. S3D). We identified *CSF3*, the gene for G-CSF, as one of the most differentially expressed genes in AML patients (Fig. 3C, Table S2). Specifically, high *CSF3* (G-CSF) gene expression was observed in AML *MLKL*^low^ patients and vice versa (Fig. 3C). This was corroborated in an independent dataset that showed a negative correlation between gene expression for *CSF3* and *MLKL* (GSE37642 and TCGA_LAML) (Fig. S3B, C).

Collectively, these data showed that high expression of *MLKL* negatively correlated with *CSF3* (G-CSF) expression.

### MLKL promotes the release of G-CSF

**Fig. 4:**
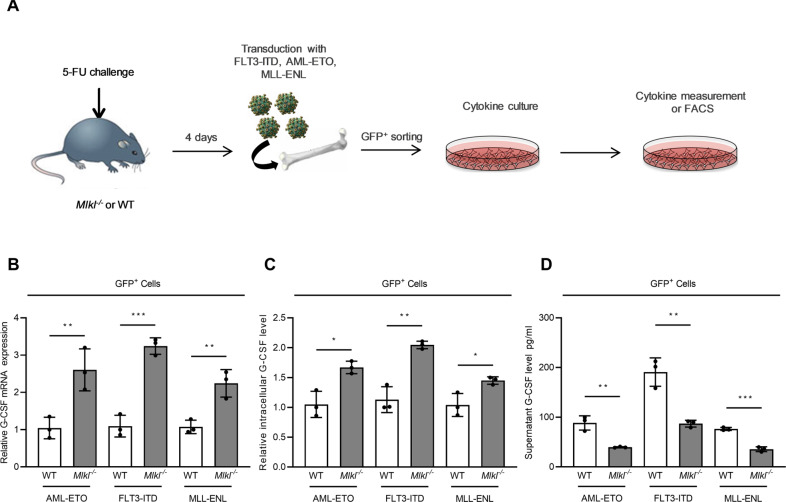
MLKL promotes the release of G-CSF. **A** Experimental design to generate leukemic BM cells transduced with oncogenic drivers AML-ETO, FLT3-ITD, or MLL-ENL together with GFP in BM cells. **B** BM cells transduced with three different oncogenes sorted (GFP^+^) and seeded with the same cell density (2 × 10^6^/ml). After 48 h cells and supernatant were collected. The mRNA expression was measured by qRT-PCR (normalized to the median of each WT group, *t*-test, mean with SD). **C** Cells as in (**B**) were measured for intracellular G-CSF level by FACS (normalized to the median of each WT group, *t*-test, mean with SD). Intensity plot in Fig. S4B. **D** Supernatant G-CSF was measured by cytometric bead array, the intensity data in Fig. S4B (*t*-test, mean with SD). *P < 0.05, **P < 0.005, ***P < 0.0005.

Inflammatory cytokines released from leukemic cells upon oncogenic signaling mediate, at least in part, the myeloid differentiation of leukemic progenitor cells [6]. Hence, we tested whether the loss of MLKL impacted the release of inflammatory mediators using primary murine leukemic progenitor cells ex vivo (Fig. 4A). Using AML-ETO, FLT3-ITD or MLL-ENL transfected cells after flow sorting to avoid cross-contamination with non-transfected progenitors, we observed that *Mlkl*^*−/−*^ AML cells expressed significantly elevated levels of *CSF3* mRNA (encoding for G-CSF) (Fig. 4B). This was interesting as G-CSF potently contributes to the proliferation and differentiation of myeloid progenitor cells [37, 38], and changes in G-CSF levels impact malignant myeloid progenitor levels in AML [39].

Next, we tested for the protein translation from *CSF3* mRNA into G-CSF protein by measuring the intracellular amount of G-CSF by FACS. We identified a significantly elevated level of G-CSF in AML cells from *Mlkl*^−*/−*^ mice when compared to WT mice (Figs. 4C, S4B). Both findings provided evidence that RNA transcription and protein translation of *CSF3* mRNA into G-CSF protein were intact and even elevated in MLKL-deficient cells.

Finally, we tested for the amount of released soluble G-CSF in the media of primary murine AML cells by FACS using supernatant from both WT and *Mlkl*^−*/−*^ cells. Interestingly, the amount of released G-CSF into the supernatant was markedly higher in WT cells when compared to *Mlkl*^−*/−*^ cells, a phenotype that was consistent across all three AML subgroups (Figs. 4D, S4B). FLT3-ITD^+^ AML cells released the highest levels of G-CSF, which is in line with our previous work showing that FLT3-ITD-induced signaling induced a substantial inflammatory response in AML progenitors (Fig. 4D) [6]. These data provided evidence that MLKL facilitates G-CSF release but did not impact *CSF3* mRNA transcription or G-CSF protein translation.

We also tested for the release of alternative cytokines other than G-CSF and detected a reduction in GM-CSF, IL-3, IL-6, and IL-1ß in individual AML models, but not consistently across all oncogenic drivers mutations (Fig. S4A).

Together, our data showed that MLKL facilitated the release of G-CSF from myeloid leukemic progenitors. *CSF3* mRNA transcription and G-CSF protein translation remained intact in MLKL-deficient cells. The negative correlation of *CSF3* and *MLKL* expression likely originates from the absence of a negative feedback loop in *Mlkl*^−*/−*^ cells as these cells fail to release equivalent amounts of G-CSF as the WT cells.

### G-CSF receptor signaling and the process of myeloid differentiation remain intact in *MLKL*-deficient cells

Based on our finding that MLKL facilitates G-CSF release, we hypothesized that MLKL influences myeloid progenitor differentiation in AML by limiting the release of G-CSF. We initially tested the ability of malignant myeloid progenitors from WT or *Mlkl*^−*/−*^ mice to undergo coordinated cellular differentiation in response to exogenous recombinant G-CSF. We cultured primary murine AML cells with recombinant murine G-CSF (mG-CSF) to activate G-CSF receptor signaling irrespective of the levels of endogenously released G-CSF. In addition, we tested the effect of an α-mG-CSF blocking antibody with respect to the inhibition of G-CSF receptor signaling. Moreover, we treated the cells with an IgG isotype to check for the extent of differentiation solely based on the presence of endogenously secreted G-CSF.

**Fig. 5:**
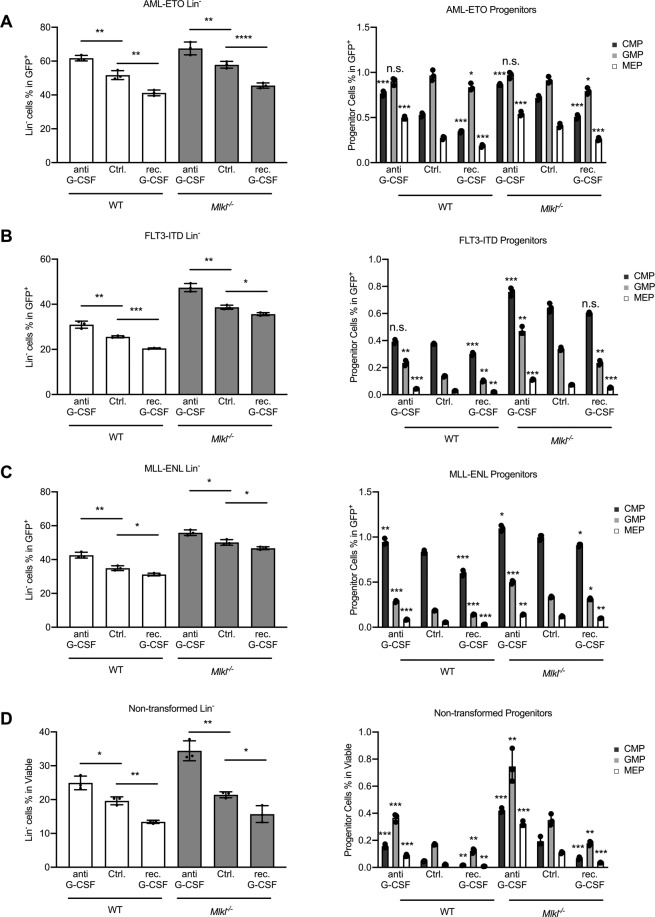
G-CSF receptor signaling and myeloid differentiation remain unaffected by the loss of MLKL. **A** Five days after oncogene transduction bone marrow cells were collected for G-CSF or anti-G-CSF treatment for another 48 h. Shown are the percentage of lineage negative cells (Lin^−^) in GFP positive cells (GFP^+^) and the percentage of progenitor cells in GFP^+^ cells from BM cells transduced with AML-ETO (*t*-test, mean with SD compared to control, FACS plots shown in Fig. S5B). **B** Treatment as in (**A**) using FLT3-ITD (*t*-test, mean with SD compared to control). **C** Treatment as in (**A**) using MLL-ENL (*t*-test, mean with SD compared to control). **D** Healthy primary murine bone marrow cells were collected and cultured for G-CSF or anti-G-CSF treatment for 48 h. Shown are the percentage of viable lineage negative cells (Lin^−^) and the percentage of viable progenitor cells (*t*-test, mean with SD). *P < 0.05, **P < 0.005, ***P < 0.0005.

We found significant repression of myeloid differentiation as measured by an expansion of primitive myeloid progenitors, when the cells were treated with α-mG-CSF antibody for all three tested AML subtypes (Figs. 5A–C, S5B). This was consistent with the notion that blocking functional G-CSF in the culture prevents myeloid differentiation, a process that proceeded independently of MLKL (Fig. 5A–D).

Next, we observed an improved myeloid differentiation as measured by loss of primitive cellular subpopulations across all three AML models when the culture was supplemented with recombinant mG-CSF in both WT and *Mlkl*^−*/−*^ genotypes (Fig. 5A–C). This finding showed that G-CSF/G-CSF-R-mediated cellular differentiation proceeded independently of MLKL when the cognate ligand for the receptor of G-CSF is available.

Irrespective of the similar pattern of response to either recombinant mG-CSF or α-mG-CSF blocking antibody in both genotypes, the number of immature myeloid progenitor cells was consistently higher in leukemic MLKL-deficient cells (Fig. 5A–C). This provided evidence that G-CSF receptor signaling remained intact in leukemic cells upon loss of MLKL. This supported the notion that MLKL specifically facilitates G-CSF release but not G-CSF receptor signaling to impact on AML differentiation.

Of note, the same pattern as described for leukemic cells was also observed in healthy primary murine BM progenitor cells when treated with recombinant mG-CSF or α-mG-CSF antibody (Fig. 5D). The loss of MLKL did not affect healthy myeloid progenitor differentiation in response to mG-CSF treatment or an α-mG-CSF antibody treatment (Fig. 5D). Consistent with our data in AML, even untransformed primary healthy murine BM cells presented with an expansion of the primitive (Lin^−^/progenitor) population (Fig. 5D), supporting the notion that MLKL facilitates G-CSF release at steady-state independent of G-CSF receptor signaling. The overall relatively small number of primitive progenitors obtained from healthy murine BM in culture was sensitive to mG-CSF or α-mG-CSF supporting the role of G-CSF in propagating myeloid progenitor differentiation (Fig. 5D).

To control whether MLKL deletion influenced the G-CSF receptor level on the cell surface, we performed FACS detecting no differences in G-CSF receptor expression (Fig. S5A).

In summary, *Mlkl*^−*/−*^ myeloid progenitors release reduced levels of G-CSF at steady-state or under conditions of oncogenic signaling. Yet, G-CSF receptor signaling remained intact despite the loss of MLKL across all three AML models and in healthy myeloid progenitors. This provided evidence that the failure of MLKL-deficient leukemic progenitors to differentiate along the myeloid lineage was, at least in part, dependent on the level of released G-CSF.

### MLKL-induced membrane permeabilization facilitates G-CSF release

MLKL contributes to plasma membrane permeabilization by a yet unknown mechanism during necroptosis [15, 40]. To understand the contribution of MLKL-induced membrane pore formation to the release of G-CSF, we co-incubated primary murine BM cells with osmotic protectors such as PEGs. PEG are hydrophilic polymers that protect against osmotic imbalance caused by the formation of membrane pores once their size restricts cytoplasmic components to pass through the pores [40–42].

**Fig. 6:**
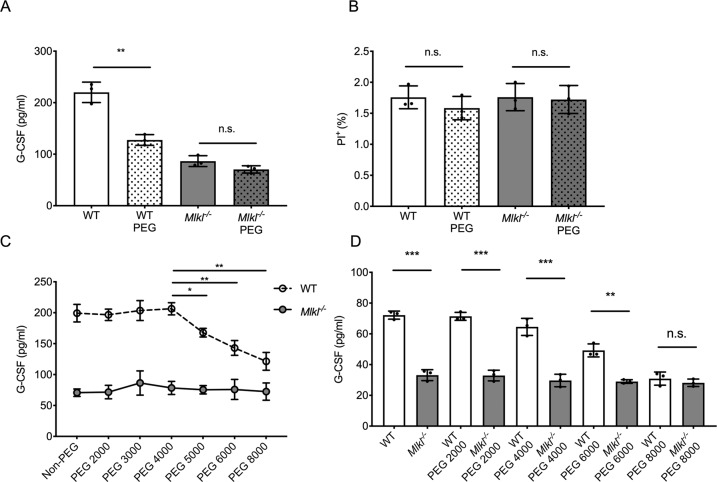
MLKL facilitates G-CSF release by controlling membrane permeabilization. **A** Release of G-CSF in WT and *Mlkl*^−*/−*^ BM cells measured in FLT3-ITD^+^ BM cells treated with or without PEG 8000 after 24 h (*t*-test, mean with SD). **B** Shown is the rate of cell death (PI^+^) of FLT3-ITD WT or *Mlkl*^−*/−*^ BM cells after 24 h of PEG8000 treatment (*t*-test, mean with SD). **C** Supernatant G-CSF levels of WT or *Mlkl*^−*/−*^ FLT3-ITD BM cells treated with PEG2000, PEG3000, PEG4000, PEG5000, PEG6000, and PEG8000 for 24 h (*P*-value between PEG4000 and higher by Student’s *t*-test). **D** Supernatant G-CSF levels of WT or *Mlkl*^−*/−*^ BM-derived macrophages (BMDMs) treated with LPS and increasing sizes of PEG for 24 h. *t*-test, mean with SD. *P < 0.05, **P < 0.005, ***P < 0.0005.

Previous studies have shown that cell death is blocked when necroptotic cells are incubated with PEG with an average molecular weight of *M*_n_ = 8000 (PEG8000). These data suggested that necroptotic pores measure approx. 4 nm in size [40]. Hence, we evaluated the effect of PEG8000 in the FLT3-ITD^+^ BM cells and found that PEG8000 effectively reduced the release of G-CSF only in WT FLT3-ITD^+^ BM cells but not in *Mlkl*^−*/−*^ FLT3-ITD^+^ BM cells (Figs. 6A, S6D). This revealed that the blockade of MLKL-induced pore formation in the plasma membrane of leukemic progenitor cells caused G-CSF release.

Of note, the levels of dying cells in culture were relatively minor and not measurably different between WT and *Mlkl*^*−/−*^ cells in the presence or the absence of PEG8000 (Figs. 6B, S6C). To test whether the effects of PEG were size-dependent, we introduced PEG (PEG2000–PEG8000) of different molecular weights with hydrated radii from 1.3 to 2 nm [41]. In FLT3-ITD^+^ WT cells, lower size PEG (PEG2000 and PEG4000) had no impact on the release of G-CSF, while larger PEG (PEG6000 and PEG8000) had a size-dependent inhibitory effect, an effect also observed in AML-ETO and MLL-ENL-transformed cells (Fig. S6E, F). None of the PEG had any effect on the *Mlkl*^−*/−*^ malignant BM cells further supporting the notion that the effect of PEG on G-CSF release was dependent on the presence of MLKL-induced pores (Figs. 6C, S6E, F). The same result was observed in non-malignant BMDM treated with lipopolysaccharide (Fig. 6D) corroborating that MLKL-induced pore formation contributed to G-CSF release in leukemic cells and also during inflammation.

Our data provide evidence that MLKL facilitates the release of G-CSF by inducing permeabilization of the plasma membrane. *Mlkl*^−*/−*^ leukemic cells inefficiently proceeded through myeloid differentiation due to reduced G-CSF release from pre-formed intracellular storage. The differentiation blockade, a hallmark of AML, is partly controlled by MLKL via a G-CSF-mediated myeloid differentiation program, which proceeds independently, or substantially before, cell death.

### MLKL contributes to G-CSF secretion via the endosomal pathway

**Fig. 7:**
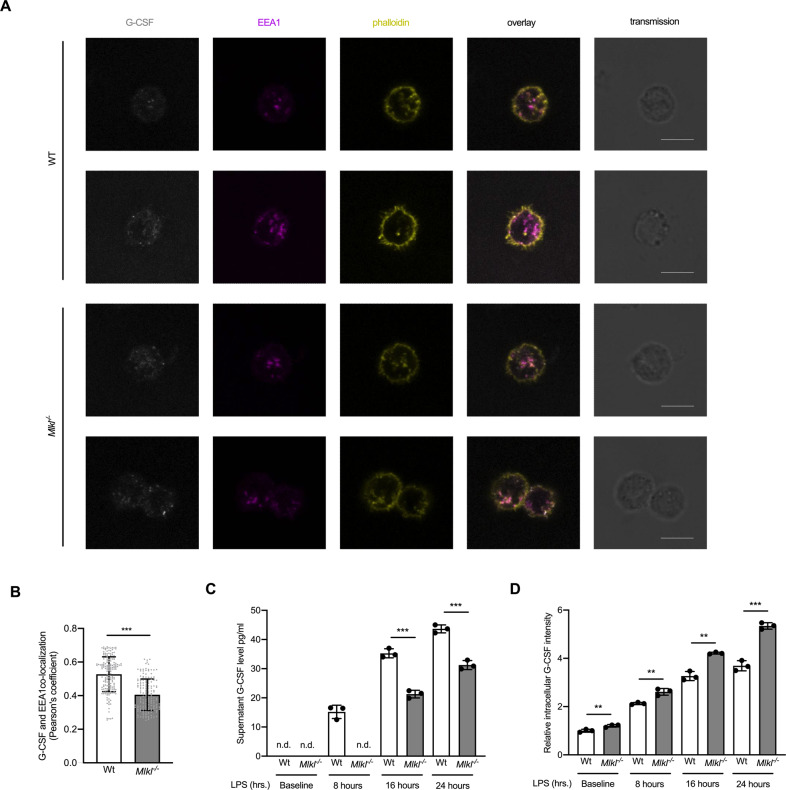
MLKL associates with early endosomal marks during endosomal trafficking. **A** Immunofluorescence images, the 5-FU challenged bone marrow cells treated with 20 ng/ml LPS for 48 h (WT or Mlkl^−*/−*^) stained as follows; gray, G-CSF; magenta, EEA1; yellow, phalloidin (which stains the plasma membrane-associated F-actin), negative and second antibody only stain FACS in Fig. S7A and B, scale bar = 100 µm. **B** Quantification of the immunofluorescence images, the co-localization of G-CSF and EEA1, Pearson’s coefficient (*t*-test, mean with SD). **C** Shown is the level of G-CSF in the supernatant of WT or *Mlkl*^−*/−*^ BM cells treated with LPS measured at serial time points (*t*-test, mean with SD). **D** Shown is the level of intracellular G-CSF in WT or *Mlkl*^−*/−*^ BM cells treated with LPS measured at serial time points (normalized to the median of the baseline WT group, mean with SD). *P < 0.05, **P < 0.005, ***P < 0.0005.

It has been reported that MLKL co-localized with endosomal marks [25, 43], influences the volume of extracellular vesicles [44], and facilitates endosomal trafficking, a reportedly cell death-independent function [25]. To explore the mechanism of how MLKL influences the secretion of G-CSF in primary murine myeloid cells, we co-stained G-CSF together with an early endosomal marker (EEA1) to quantify the distribution of G-CSF and the endosomal compartment using confocal microscopy. To obtain a sufficiently high resolution of G-CSF for microscopy, the cells were stimulated with LPS prior to the analysis, which triggers G-CSF production and secretion [45]. One day after treatment, murine BM cells were stained with EEA1, G-CSF, and phalloidin. We observed a significantly reduced co-localization of G-CSF with EEA1 in *Mlkl*^−*/−*^ cells as compared to WT cells while the intercellular EEA1 level remains similar between WT and *Mlkl*^−*/−*^ cells (Figs. 7A, B, S7C) suggesting that loss of MLKL prevented G-CSF to efficiently enter the endosomal compartment.

As a previous publication reported *Mlkl* deletion to slow down the trafficking of membrane-associated proteins [25], we examined this aspect over serial time points using LPS-treated BM cells and testing for secreted G-CSF levels in the supernatant. We found an overall reduced and delayed propensity of *Mlkl*^*−/−*^ cells to release G-CSF (Fig. 7C). This reduction markedly contrasts with the intracellular G-CSF protein levels in *Mlkl*^*−/−*^ cells at baseline and at any time point during the experiment (Fig. 7D).

The accumulation of *CSF3* mRNA (Fig. 4B) and G-CSF intracellular protein (Figs. 4C, 7D), the dependency on MLKL-induced pore formation (Fig. 6) together with the failure to efficiently release G-CSF from intracellular storage (Fig. 7C, D), supported the notion that a negative feedback mechanism keeping G-CSF protein production in check was ineffective in *Mlkl*^*−/−*^ cells resulting in the observed phenotype.

In summary, MLKL facilitates the secretion of G-CSF by controlling endosomal trafficking. Accordingly, MLKL-deficient leukemic cells inefficiently proceed through myeloid differentiation due to reduced G-CSF secretion and subsequent myeloid differentiation.

## Discussion

MLKL serves as a critical mediator of necroptotic cell death by inducing the formation of membrane pores upon phosphorylation by RIPK3 [13, 15, 17]. As RIPK3 functions as a powerful tumor suppressor in myeloid leukemia [6], we set out to investigate the contribution of its downstream partner MLKL to leukemogenesis. Previously, we reported that MLKL restricted leukemogenesis, however, the molecular mechanism of its contribution remained unclear [6], specifically since *Mlkl*^*−/−*^ leukemic cells remained capable of undergoing cell death [6].

Constitutive signaling from oncogenic drivers in AML provides a pathophysiologically relevant cell death stimulus to leukemic cells [6]. This is caused by pro-inflammatory gene expression, which co-activates RIPK3-dependent cell death and represses leukemogenesis [4, 6]. Subsequently, leukemic cells in AML patients repress RIPK3 or alternative members of the pathway to secure their continued survival. Despite the contribution of MLKL to plasma membrane pore formation, its contribution to cell death in leukemic cells remains less well-defined. This is based on the finding that cell death in response to oncogenic signaling proceeded independently of MLKL (Fig. 6B, S2A–C), which argues that MLKL-induced plasma membrane pore formation in AML, albeit involved in cytokine release, did not fully translate into cell death.

Published work on the endosomal sorting complex required for transport (ESCRT) in necroptosis, specifically ESCRT-III, supports this finding by showing that ESCRT-III sustains the plasma membrane integrity of cells when MLKL activation is incomplete or limited by alternative factors [24]. We, therefore, speculate that MLKL-induced pore formation is kept in check by ESCRT or alternative factors that restrict its full activation by controlling the number, the timing, or, alternatively, the size of the pores. A molecular mechanism keeping pore formation in check has been described for several scenarios in which pore-forming proteins or toxins can be counterbalanced to allow for the continued survival of the affected cells [46–50].

In leukemic cells, the amount of activated MLKL in response to oncogenic signaling [6] might be below a threshold for cell death induction, which suffices for the release of pre-formed G-CSF. A study by Ros and colleagues estimated that the pore size of necroptotic pores measures around 4 nm [40], which would allow the passage of G-CSF molecules into the supernatant. Our data using PEG evidently support this notion suggesting that pore formation not necessarily proceeds to cell death and reasoning that protective factors might prevent leukemic cells from cell death/necroptosis. The identification of such protective factors might serve as a powerful tool to induce cell death in AML pharmaceutically if inhibiting agents became available.

Additional functions of MLKL have been reported that are not primarily based on cell death induction. These include MLKL in endosomal trafficking [25], insulin sensitivity, and type II diabetes [51] or the requirement for MLKL for myelin sheath breakdown [52]. In the light of published reports and our data, we cannot fully exclude the possibility that very low levels of cell death that remain at or below the detection limit of our assays suffice to increase the G-CSF levels in the supernatant to a degree relevant for myeloid differentiation.

Additional support for a role of MLKL in cytokine release comes from a study by Yoon et al. that reported that deletion of *MLKL* resulted in a significant upregulation of mRNA of cytokines such as *CSF1* (encoding M-CSF), *CSF2* (encoding GM-CSF), and *IL-8* amongst others [25], Using the same markers for detection of early endosome formation (EEA1) that were reported by Yoon et al., we identified a very similar reduction in endosomal trafficking in our study. Whereas *CSF3* (G-CSF) was not reported in their study, the data supported the notion that low/reduced levels of MLKL contribute to a reduction in released cytokine levels and a subsequent failure of a negative feedback mechanism keeping cytokine mRNA expression in check [25]. A publication showing that MLKL controls intracellular membranes and inhibits autophagy during necroptosis also supports a possible role in membrane physiology [18].

It is interesting to note that the main differences in cytokine release were observed for G-CSF in our study, whereas alternative cytokines such as TNF or IL-6 differed to a lower extent. The type of oncogenic driver, the time points of analysis, or the propensity of primary murine BM-derived cells to release G-CSF might cause this difference. Irrespective of alternative cytokines, our data support a critical contribution to MLKL-mediated G-CSF release for the process of cellular differentiation in AML. This is of specific interest as we utilized three clinically relevant AML oncogenic drivers that together represent the majority of cytogenetically normal AML patients.

A substantial body of evidence shows that G-CSF instructs lineage choice and proliferation of hematopoietic progenitor cells during steady-state hematopoiesis [53–55], as well as during emergency granulopoiesis [53, 56, 57]. Yet, the application of recombinant G-CSF as a therapeutic agent in clinical hematology in AML patients is often considered with a certain reservation. This is due to the potentially pro-proliferative effect of G-CSF on leukemic stem and progenitors cells and a possible disease propagation. The use of recombinant G-CSF in AML patients is therefore often restricted to patients in molecular remission or for situations of dire clinical need such as severe infections and sepsis. However, we consider it reasonable to speculate that the activation of myeloid differentiation using recombinant G-CSF as a therapeutic agent in AML might provide a clinical benefit to AML patients, specifically for those that express low MLKL. Patients diagnosed with acute promyelocytic leukemia (APL) represent a prime example of the therapeutic benefit of differentiation-inducing agents in AML [58, 59].

Instead of using a loss-of-function model of MLKL as in our study, Hildebrand et al. recently reported that an auto-active mutant of MLKL (*Mlkl*^*D139V*^), elevated the plasma levels of several pro-inflammatory cytokines (including G-CSF) [60]. The report shows elevated CD45^+^ cellular infiltrates in *Mlkl*^*D139V*^/^*D139V*^ mice, yet no clear correlation to exaggerated cell death [60]. It is reasonable to speculate that the alterations to the hematopoietic system might stem from elevated cytokine release from overactive MLKL supporting the function of MLKL in facilitating cytokine release.

In summary, we show that the differentiation blockade in AML partly results from the failure of AML cells to release G-CSF from intracellular storage. Our findings thereby expand the molecular mechanism of differentiation blockade in AML to auto- or paracrine G-CSF-mediated signaling. Our data provide a pathophysiological context for the observation that pore formation in the plasma membrane and/or endosomal trafficking can be blocked or delayed without necessarily proceeding to cell death.

## Supplementary information

Supplementary legends

Supplementary Figure S1

Supplementary Figure S2

Supplementary Figure S3

Supplementary Figure S4

Supplementary Figure S5

Supplementary Figure S6

Supplementary Table S1

Supplementary Figure S7

Supplementary Table S2

Supplementary Table S3

Supplementary Table S4
