# Correction: MLKL promotes cellular differentiation in myeloid leukemia by facilitating the release of G-CSF

**DOI:** 10.1038/s41418-021-00826-8

**Published:** 2021-06-30

**Authors:** Xin Wang, Uris Ros, Deepti Agrawal, Eva C. Keller, Julia Slotta-Huspenina, Veronika Dill, Bo Shen, Run Shi, Tobias Herold, Claus Belka, Ritu Mishra, Florian Bassermann, Ana J. Garcia-Saez, Philipp J. Jost

**Affiliations:** 1 Medical Department III for Hematology and Oncology, School of Medicine, Klinikum rechts der Isar, Technical University of Munich, Munich, Germany; 2 Department of Internal Oncology, Jiangsu Cancer Hospital (Nanjing Medical University Affiliated Cancer Hospital) and Jiangsu Institute of Cancer Research, Nanjing, China; 3 Institute for Genetics, Faculty of Mathematics and Natural Sciences, Universität zu Köln, Cologne, Germany; 4 Institute of Pathology, School of Medicine, Klinikum rechts der Isar, Technical University of Munich, Munich, Germany; 5 Department of Radiation Oncology, University Hospital, Ludwig-Maximilians University, Munich, Germany; 6 Laboratory for Leukemia Diagnostics, Department of Medicine III, University Hospital, LMU Munich, Munich, Germany; 7 Research Unit Apoptosis in Hematopoietic Stem Cells, Helmholtz Zentrum München, German Research Center for Environmental Health (HMGU), Munich, Germany; 8 German Consortium for Translational Cancer Research (DKTK) partner site TUM, German Cancer Research Center Heidelberg (DKFZ), Heidelberg, Germany; 9 Center for Translational Cancer Research, Translatum, Technical University of Munich, Munich, Germany; 10 Division of Clinical Oncology, Department of Medicine, Medical University of Graz, Graz, Austria

**Keywords:** Cancer, Cancer

Correction to: *Cell Death & Differentiation* 10.1038/s41418-021-00811-1, published online 2 June 2021

The original version of this article unfortunately contained a mistake in an author name. Dr. Ritu Mishra was misspelled as “Misra”. The authors apologize for the mistake. The original article has been corrected.

