# Supplementary material for: MLKL promotes cellular differentiation in myeloid leukemia by facilitating the release of G-CSF
Source: Cell Death Differ. 2021 Jun 2;28(12):3235–50. doi: 10.1038/s41418-021-00811-1 (PMC8630008; doi:10.1038/s41418-021-00811-1)
Supplement: Supplementary file 1 — Supplementary legends [file 41418_2021_811_MOESM1_ESM.docx]

# **SUPPLEMENTARY FIGURE LEGENDS**

# **MLKL promotes cellular differentiation in myeloid leukemia by facilitating the release of G-CSF**

## **Supplementary Figure S1 related to Figure 1.**

A. cBioPortal for Cancer Genomics (http://cbioportal.org) was queried for mutation frequency of *MLKL* in different cancers (date: 09/2019) using bar graphs to display the frequency.

B. Lolli plot visualizing the distribution of *MLKL* alterations identified in various cancers within the protein sequence of *MLKL* as analyzed by cBioportal.

## **Supplementary Figure S2 related to Figure 2.**

A-C. Shown is the viability of BM cells (WT and *Mlkl^-/-^*) transduced with (A) AML-ETO, (B) FLT3-ITD or (C) MLL-ENL linked to Figure 2A-C (t-test, mean with SD).

D. Shown are gating strategies and progenitor populations in GFP**^+^** cells in AML-ETO, FLT3-ITD or MLL-ENL (t-test, mean with SD) linked to Figure 2A-F.

*P<0.05, **P<0.005, ***P<0.0005

## **Supplementary Figure S3 related to Figure 3.**

A. Enrichment plots for gene set enrichment analysis (GSEA) linked to Figure 3.

B and C. Shown is the correlation between *CSF3* and *MLKL* mRNA expression in GSE37642 (n=422) and TCGA_LAML datasets (n=173) (Pearson correlation).

D. qPCR results for the most differential expressed genes in Figure 3C, between WT and *Mlkl^-/-^* bone marrows cross AML-ETO, FLT3-ITD and MLL-ENL oncogenes (t-test, mean with SD).

*P<0.05, **P<0.005, ***P<0.0005

## **Supplementary Figure S4 related to Figure 4.**

A. Shown are supernatant cytokine measurements for GM-CSF, IL-3, IL6, TNF and IL-1β linked to Figure 4D (t-test, mean with SD).

B. Shown are histograms of cytokines measured in BM cells linked to Figure 4C-D, red represent signal from *Mlkl^-/-^* group, blue represent signal from WT group.

*P<0.05, **P<0.005, ***P<0.0005

## **Supplementary Figure S5 related to Figure 5.**

A. The relative surface GCSFR level were measured between WT and *Mlkl^-/-^* bone marrows cross AML-ETO, FLT3-ITD and non-transformed bone marrow cells with surface stain FACS (t-test, mean with SD).

B. Shown are gating strategies and progenitor populations in BM cells using AML-ETO-transduced cells as example after treatment with α-G-CSF antibody or rec. G-CSF (t-test, mean with SD) linked to Figure 5.

*P<0.05, **P<0.005, ***P<0.0005

## **Supplementary Figure S6 related to Figure 6.**

A and B, BMDMs treated with high level of LPS (100ng/ml which could induce cell death) for 24 hours, the PI^+^ and supernatant G-CSF level were measured by FACS when treated with or without PEG 8000 (student´s t-test, mean with SD).

C & D, BMDMs treated with low level of LPS (20ng/ml which could not induce cell death) for 24 hours, the PI^+^ and supernatant G-CSF level were measured by FACS when treated with or without PEG 8000 (mean with SD).

E and F, Supernatant G-CSF levels of AML-ETO and MLL-ENL WT or *Mlkl^-/-^* BM cells treated with PEG2000, PEG3000, PEG4000, PEG5000, PEG6000 and PEG8000 for 24 hours. P-value between PEG5000 and PEG6000 or PEG8000 (P-value between PEG4000 and higher by student´s t-test).

*P<0.05, **P<0.005, ***P<0.0005

## **Supplementary Figure S7 related to Figure 7.**

A and B. negative and single 2^nd^ antibody satin related to Figure 6A.

C. the relative EEA1 intensity related to Figure 6A (t-test, mean with SD).

*P<0.05, **P<0.005, ***P<0.0005

# **SUPPLEMENTARY TABLE LEGENDS**

## **Table S1. FDR P values for heatmap in Figure 3C**

FDR P values of top 50 differentially expressed genes in inflammatory response pathway from Figure 3C.

## **Table S2. Data for the heatmap in Figure 3C**

TCGA_LAML data for making the heatmap in Figure 3C, unit is log_2_(RSEM counts+1).

## **Table S3. Primer list**

Primer list for qPCR.

## **Table S4. Antibody list**

Antibody list.
