# Supplementary figures and images for: MLKL promotes cellular differentiation in myeloid leukemia by facilitating the release of G-CSF
Source: Cell Death Differ. 2021 Jun 2;28(12):3235–50. doi: 10.1038/s41418-021-00811-1 (PMC8630008; doi:10.1038/s41418-021-00811-1)

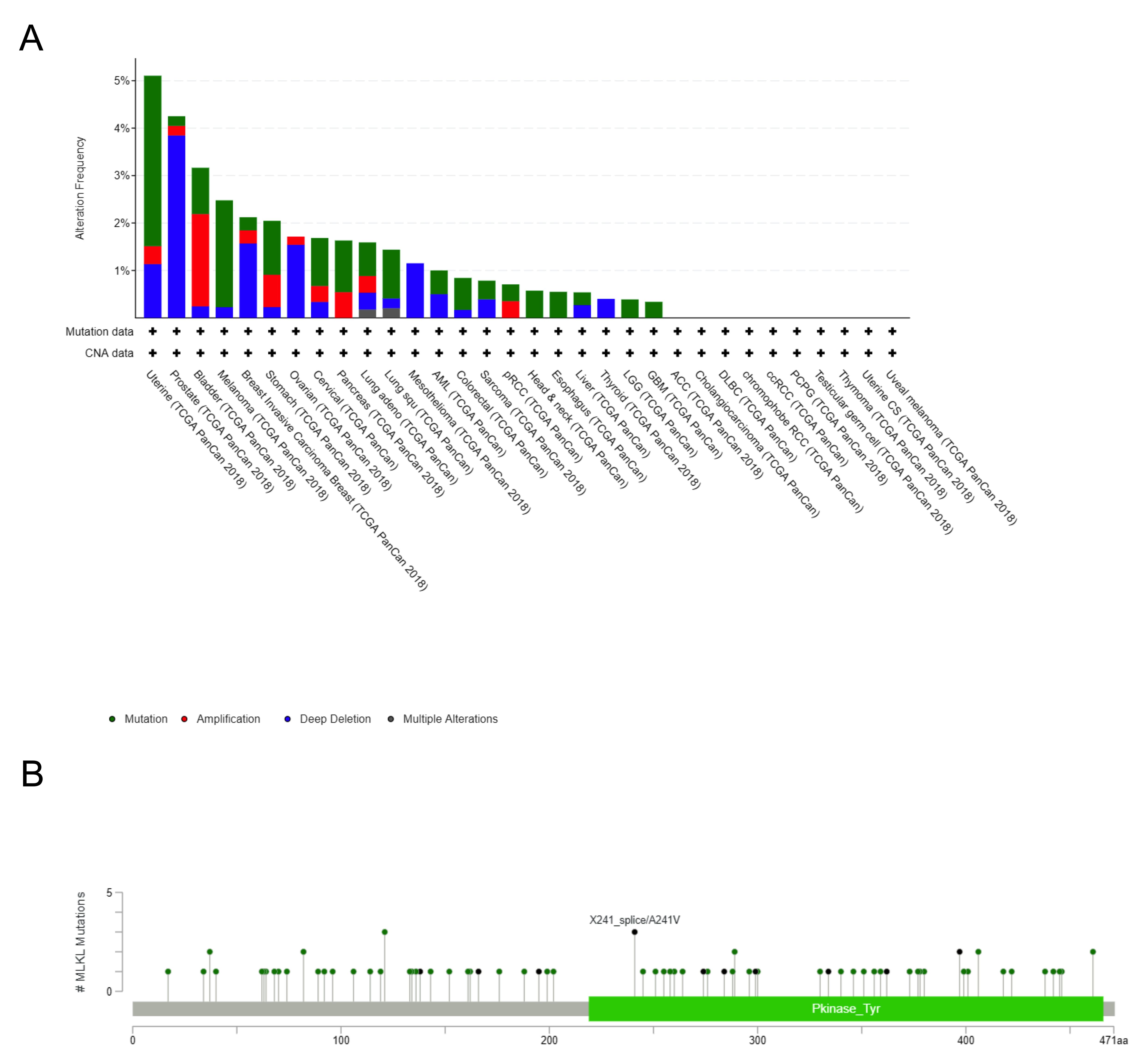

Supplement: Supplementary file 2 — Supplementary Figure S1 [file 41418_2021_811_MOESM2_ESM.tif]

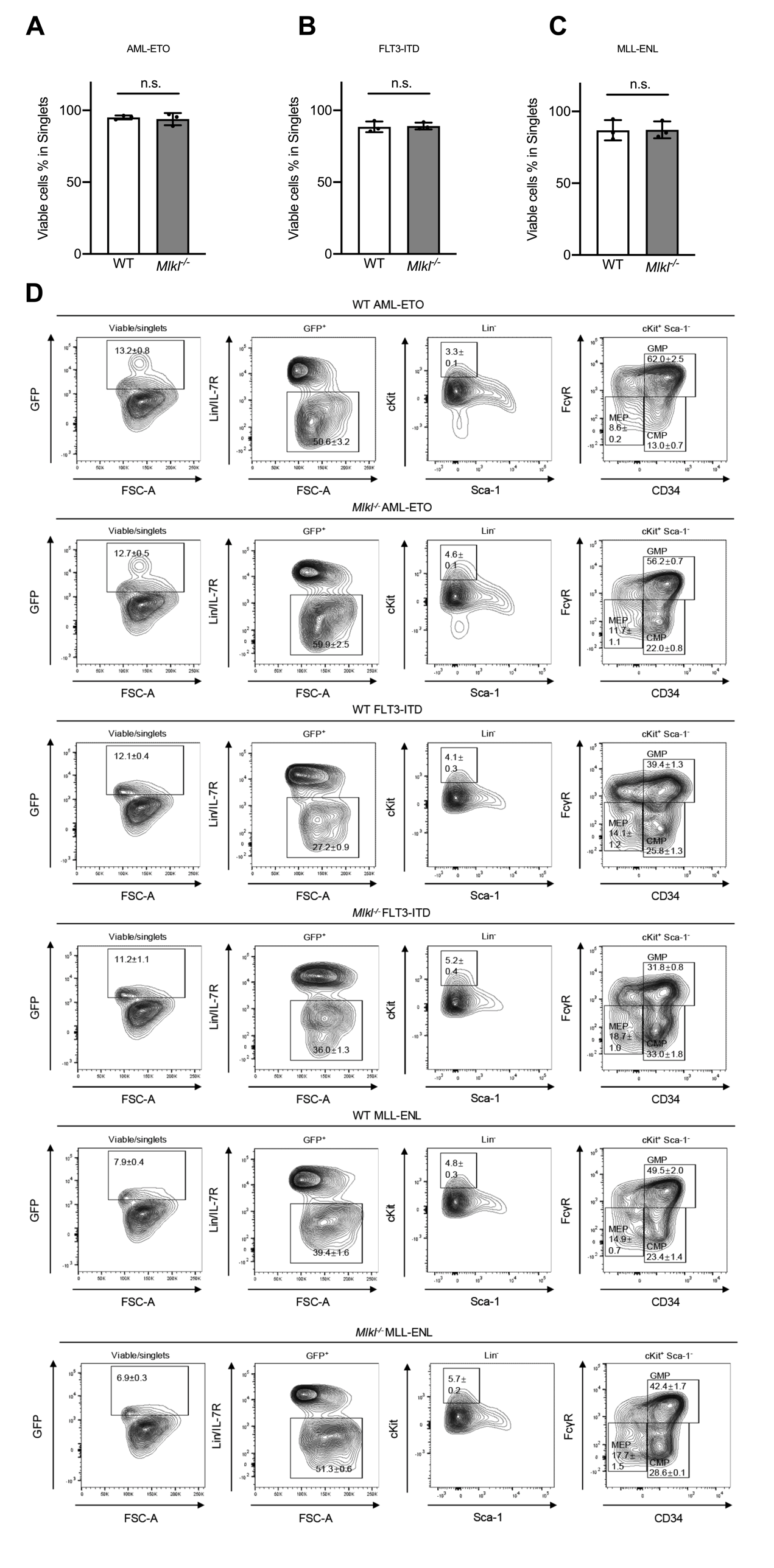

Supplement: Supplementary file 3 — Supplementary Figure S2 [file 41418_2021_811_MOESM3_ESM.tif]

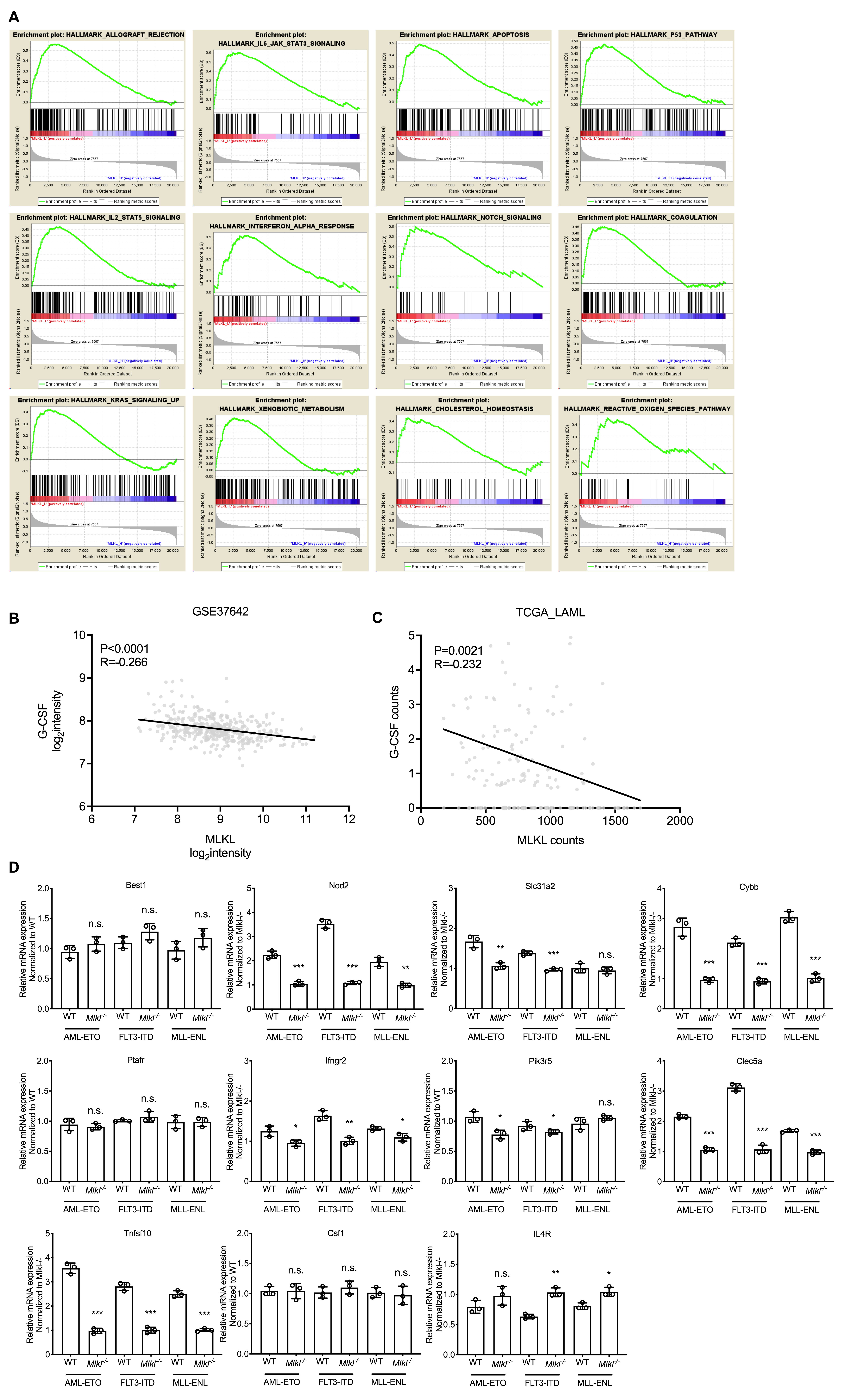

Supplement: Supplementary file 4 — Supplementary Figure S3 [file 41418_2021_811_MOESM4_ESM.tif]

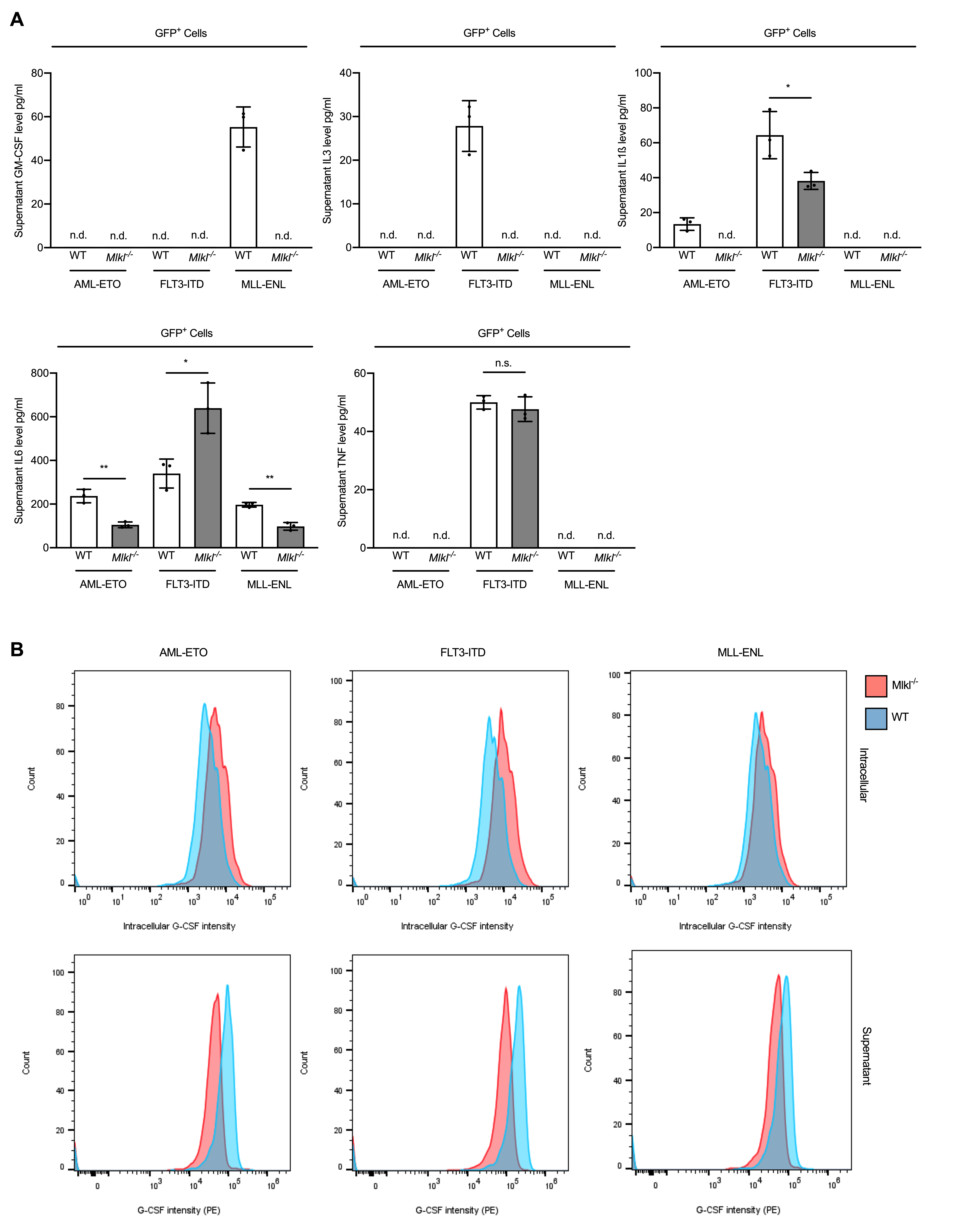

Supplement: Supplementary file 5 — Supplementary Figure S4 [file 41418_2021_811_MOESM5_ESM.tif]

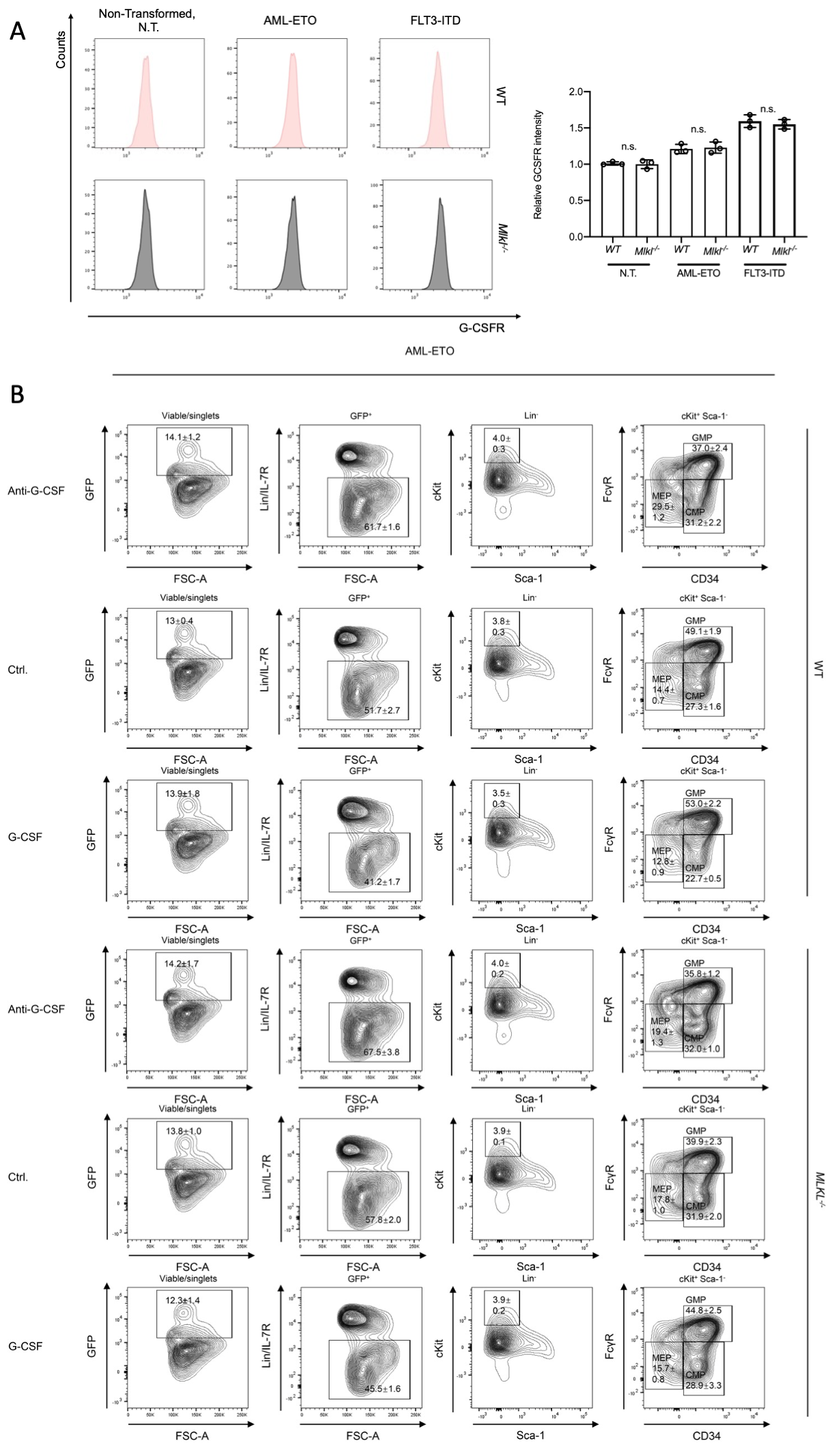

Supplement: Supplementary file 6 — Supplementary Figure S5 [file 41418_2021_811_MOESM6_ESM.tif]

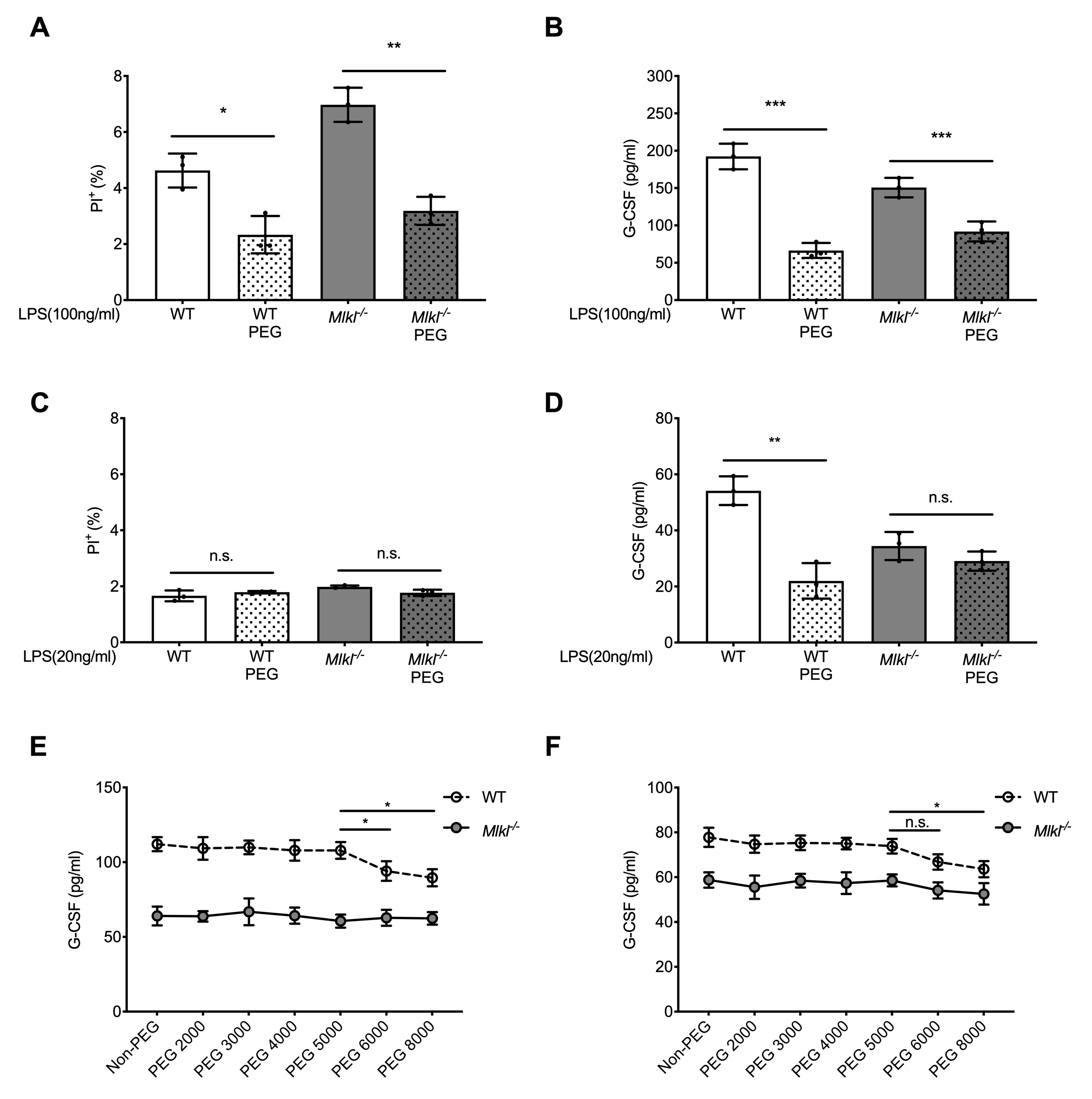

Supplement: Supplementary file 7 — Supplementary Figure S6 [file 41418_2021_811_MOESM7_ESM.tif]
